# Stair Gait in Older Adults Worsens With Smaller Step Treads and When Transitioning Between Level and Stair Walking

**DOI:** 10.3389/fspor.2020.00063

**Published:** 2020-06-25

**Authors:** Irene Di Giulio, Neil D. Reeves, Mike Roys, John G. Buckley, David A. Jones, James P. Gavin, Vasilios Baltzopoulos, Constantinos N. Maganaris

**Affiliations:** ^1^Centre for Human and Applied Physiological Sciences, King's College London, London, United Kingdom; ^2^Research Centre for Musculoskeletal Science and Sports Medicine, Department of Life Sciences, Manchester Metropolitan University, Manchester, United Kingdom; ^3^Rise and Going Consultancy, Watford, United Kingdom; ^4^School of Engineering, University of Bradford, Bradford, United Kingdom; ^5^School of Health Sciences, University of Southampton, Southampton, United Kingdom; ^6^Research Institute for Sport and Exercise Sciences, Liverpool John Moores University, Liverpool, United Kingdom

**Keywords:** stair negotiation, balance control, step going, fall risk, old people

## Abstract

Older people have an increased risk of falling during locomotion, with falls on stairs being particularly common and dangerous. Step going (i.e., the horizontal distance between two consecutive step edges) defines the base of support available for foot placement on stairs, as with smaller going, the user's ability to balance on the steps may become problematic. Here we quantified how stair negotiation in older participants changes between four goings (175, 225, 275, and 325 mm) and compared stair negotiation with and without a walking approach. Twenty-one younger (29 ± 6 years) and 20 older (74 ± 4 years) participants negotiated a 7-step experimental stair. Motion capture and step-embedded force platform data were collected. Handrail use was also monitored. From the motion capture data, body velocity, trunk orientation, foot clearance and foot overhang were quantified. For all participants, as stair going decreased, gait velocity (ascent p_A_ = 0.033, descent p_D_ = 0.003) and horizontal step clearance decreased (p_A_ = 0.001), while trunk rotation (p_D_ = 0.002) and foot overhang increased (p_A,D_ < 0.001). Compared to the younger group, older participants used the handrail more, were slower across all conditions (p_A_ < 0.001, p_D_ = 0.001) and their foot clearance tended to be smaller. With a walking approach, the older group (*Group x Start* interaction) showed a larger trunk rotation (p_A_ = 0.011, p_D_ = 0.015), and smaller lead foot horizontal (p_A_ = 0.046) and vertical clearances (p_D_ = 0.039) compared to the younger group. A regression analysis to determine the predictors of foot clearance and amount of overhang showed that physical activity was a common predictor for both age groups. In addition, for the older group, medications and fear of falling were found to predict stair performance for most goings, while sway during single-legged standing was the most common predictor for the younger group. Older participants adapted to smaller goings by using the handrails and reducing gait velocity. The predictors of performance suggest that motor and fall risk assessment is complex and multifactorial. The results shown here are consistent with the recommendation that larger going and pausing before negotiating stairs may improve stair safety, especially for older users.

## Introduction

Aging is a progressive process in which the physical and cognitive abilities deteriorate (Lord et al., 1996; Startzell et al., 2000), with a negative effect on motor performance and confidence whilst performing daily activities. Gait problems are common in old age (Lord et al., 1996; Jahn et al., 2010), and falls are usually associated with some deficits in the locomotor ability (Prince et al., 1997; Begg and Sparrow, 2000). Every year, 1/3 of individuals over 65 years old experience a fall (World Health Organisation, 2007). Indeed, falls are a major cause of morbidity in older people and the primary cause of accidental death (World Health Organisation, 2007; Age, 2012). Older people may experience difficulties because their locomotor pattern can become less efficient, in addition to impairments in their adaptive and recovery mechanisms (Rogers et al., 2003). Gait on stairs is a key example of this difficulty: the task is not only constrained (see below), it also places additional demands on the musculoskeletal and balance control systems, compared to level walking (McFadyen and Winter, 1998; Startzell et al., 2000; Riener et al., 2002; Reeves et al., 2008, 2009). Not surprisingly, a large number of dangerous falls occur during stair negotiation (Svanstrom, 1974; Jacobs, 2016).

Although the recommendations for stair rise height in private and public buildings are specific (170–220 mm), the UK guidelines prescribe goings (i.e., the horizontal distance between two consecutive step edges) between 220 and 400 mm (Government, 2010), which highlights large variation in recommendation. Stair going determines the antero-posterior area for foot placement and stride length, which are critical aspects for locomotion safety and fall risk. For stair safety, going dimensions should allow safe foot placement in descent, reducing foot overhang (that is, the portion of the foot that is not placed on the stair step), and allow the individual to develop an adequate push-off in ascent to propel the body upwards, increasing foot clearance (that is, the distance between edge of the foot and stair step). These are particularly important for older people because they may be less able to react if foot placement is not optimal, and they may be less able to lift the foot to clear a step as their strength reserves may be lower. Changing step going could improve safety on stairs for users, and older individuals specifically. However, understanding the motor adaptations in relation to intact and impaired balance performance is necessary before stricter guidelines can be suggested. For example, stride length is often adapted in level walking (Patla, 2003) in response to deterioration of neuro-musculo-skeletal health, balance ability and general physical wellness. However, stride length is constrained by the step dimensions on stairs. This is more problematic for sedentary older people because their declined neuromuscular and cardiovascular capacities are taxed by stair negotiation which requires moving the body center of mass forward and upward, against gravity (in ascent), and controlling balance and accurate foot placement, especially in descent.

Furthermore, a less efficient locomotor pattern can affect stair negotiation when preceded or followed by level gait. With a walking approach, the nervous system has to quickly respond to a change in the motor task and produce a quasi-feedforward programme (Patla, 2003). As the central and peripheral nervous system may become less efficient with aging, with impaired proprioception and worsened reaction times (Rogers et al., 2003), the need to change a motor programme quickly may introduce an additional motor control difficulty. Taken together, the neuro-musculo-skeletal difficulties and the constraints and demands imposed by stair negotiation could explain why older people are more prone to problems and accidents on stairs (Cavanagh et al., 1997).

**Figure 1:**
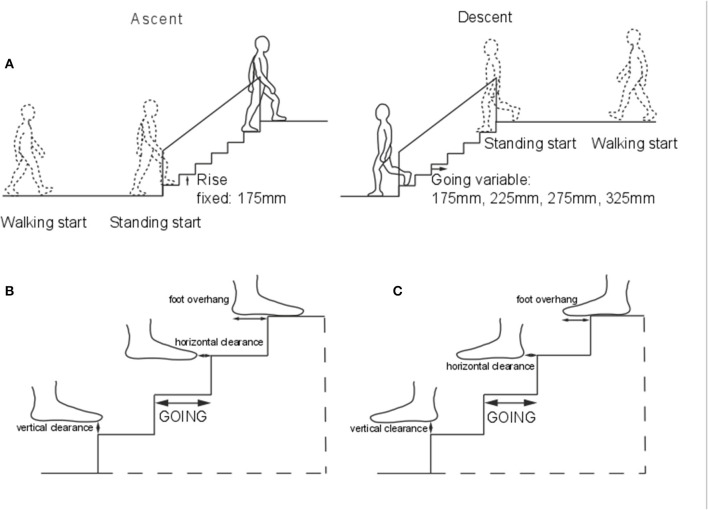
Apparatus. **(A)** The seven-step instrumented stair. Step size: rise (height) 175 mm, going 175, 225, 275, and 325 mm. Four force platforms were embedded in steps 2–5. Handrails were provided on each side. Representation of the vertical and horizontal clearance and overhang in ascent **(B)**, and descent **(C)**.

In this study, we investigated the effect of changing the going (Figure 1) on stair negotiation performance and safety, by measuring key parameters including body orientation, velocity, foot clearance and overhang. We asked: (i) Does going size affect stair negotiation in older, more than in younger participants? (ii) Is the difference in performance of older and younger participants amplified in the case of a transition in motor tasks (i.e., level walking and stair negotiation)?

## Methods

### Ethical Approval

Participants gave written informed consent to these experiments, which conformed to the Declaration of Helsinki and were approved by the ethics committee of the Institute for Biomedical Research into Human Movement and Health, Manchester Metropolitan University.

### Participants and Procedure

Twenty-one young (thirteen men, eight women; mean ± standard error “SE” 29 ± 1 years; mass 77.2 ± 4.7 kg; height 1.75 ± 0.003 m) and 20 older participants (10 men, 10 women; 74 ± 1 years; 75.2 ± 4.3 kg; 1.66 ± 0.003 m) negotiated a stair (Figure 1A) at their self-selected speed. All participants were healthy and were recruited from the local community. Participants were included if they did not report musculoskeletal, neurological or cardiovascular pathologies, which would make stair negotiation risky. Participants were barefoot to minimize the influence of footwear on performance and on walking speed (Menz et al., 2003a), socks were not allowed to standardize friction between the stair and feet. The experiment was performed in a well-lit laboratory, with natural light from windows, and artificial light available when needed, but ambient light level was not controlled. Before the current protocol, each participant performed at least five stair negotiations on a different stair for familiarization. Before the session, participant's left and right leg and foot lengths were measured.

Participants performed four trials in a randomized order: ascent and descent from standing start, and ascent and descent preceded and followed by walking on a 2 m-walkway. Step going of the stair was also randomized for the four goings tested (175, 225, 275, and 325 mm). The experimental staircase was designed without protruding nosings. Stair rise was set at 175 mm, which is within the current recommendations for stair in private and public buildings (170–220 mm) (Government, 2010).

### Apparatus and Measurement

As the average foot size is 260 mm, an adjustable seven-step stair was used with the following going sizes: small that restricted whole foot placement (175 mm), current standard for domestic stairs (225 mm), standard for semi-public buildings (275 mm), and standard for public buildings that allowed comfortable whole foot placement (325 mm) (Roys, 2001).

The stair had four 300 × 500mm force platforms (model 9260AA3, Kistler Instrumente, CH-8408 Winterthur, Switzerland) embedded in the second, third, fourth and fifth steps. The force platforms were used to determine when the foot landed and lifted-off the step. Handrails were provided on both sides of the stair. A safety-harness system suspended from a trolley and girder on the ceiling of the laboratory was secured to the participant. The stair was situated in a volume covered by a 10-camera optoelectronic movement analysis system (Vicon Motion Systems, Oxford, UK). Retro-reflective markers (14 mm) were attached to the participant's skin or tight-fitting clothes at landmarks according to the Plug-In-Gait model, with additional markers on the fifth metatarsal head, the dorsal aspect of the second toe distal tip, on the lateral and medial aspects of the calcaneus and the medial malleoli. Kinematic data were collected at 100 Hz.

As the stair protocol lasted for about 3 h, further data was collected on a second visit. This data included fear of falling questionnaire, although here only an overall score is reported (0 = no fear, 5 = very high fear), self-reported hours of physical activity per week, and total medications taken (Lord et al., 2007). Three tests measured participants' balance using the ground reaction force data sampled at 1,000 Hz (AMTI, OR6-7, Watertown, MA, USA): (1) Standing on the self-selected leg with eyes open for 5 s (used to probe and exacerbate the balance challenges of the single support phase in stair negotiation), (2) quiet standing for 30 s with eyes open (EO), and (3) with eyes closed (EC).

### Data Analysis

Each stair trial was visually inspected offline to record handrail use, body orientation and stepping method. Trials were initially assigned a nominal 0 for these indexes. If the participant touched one or both handrails, the trial was given a nominal value of 1 for handrail use. If the body was orientated toward one handrail, the trial was given a nominal value of 1 for change in orientation. If the participant placed both feet on one step, the trial was given a nominal value of 1 for change in stepping method.

The following quantities were calculated using Matlab scripts (Mathworks, Natick, US).

*Mean Gait Velocity*. The mean antero-posterior velocity of the center of mass of the upper body (trunk and head) over the whole stair. The 3D center of the upper body (four head markers, 7th cervical vertebra, 10th thoracic vertebra, right scapula, sternum and clavicle notch) was determined. The antero-posterior component of its position was extracted and differentiated to compute the velocity. The upper body was chosen to represent gait velocity, because the markers used were less affected by camera visibility obstruction from the stair in the large volume captured.

*Trunk Orientation*. The angle between the trunk antero-posterior axis and the direction of travel (from the laboratory coordinates), at foot landing (when the force plate signal first crossed a 10N threshold) in the horizontal plane. The average at steady-state (steps with force plates, 2–5) relative to the initial orientation of the trunk when the person was standing still (0–500 ms) was calculated. 0deg indicates no change in trunk orientation.

*Foot Overhang*. The antero-posterior foot portion landing outside the step (Figures 1B,C) as a percentage of the antero-posterior foot length on the step at steady-state (see above). The fore-foot was identified as the geometrical average of the markers placed on the second and fifth metatarsal head and the dorsal aspect of the second toe distal tip. The rear-foot was identified as the geometrical average of the markers placed on the heel and the lateral and medial aspects of the calcaneus. Markers' size was accounted for in the overhang calculations. The coordinates of the step edges were included in the algorithm for the calculations, based on the force platforms positions, included in the motion capture software. Negative values indicate overhang. Left and right feet overhang were averaged to provide a mean per trial.

*Foot Clearance*. The minimum distance between fore- and rear-foot (as calculated for foot overhang in ascent and descent, respectively, Figures 1B,C) and each step edge during swing, in the horizontal and vertical direction and for the lead (landing on the step) and trail limb (landing on the following step). The coordinates of the step edges were included in the algorithm for the calculations, based on the force platforms positions, included in the motion capture software. Clearances were calculated for each step and the average over the central steps (2–5) for each of the four clearances were also calculated. From the individual's step clearance, a coefficient of variation was calculated as an indicator of the repeatability and precision of foot placement.

For the balance tests, the Center of Pressure (CoP) was measured from the point of application of the ground reaction force to evaluate body sway. To evaluate balance abilities we calculated:

*Single-Leg Balance*. The root mean square (RMS) medio-lateral deviation of the CoP. A lower value indicates better control of balance.

*Balance With Eyes Closed vs. Eyes Open (EC vs. EO)*. The ratio between the antero-posterior RMS CoP from the eyes closed and eyes open tests. A ratio >1 means a higher sway in the eyes closed condition.

### Statistical Analysis

For all the statistical tests significance was set at *p* ≤ 0.05. Results are reported as mean ± SE. Stair ascent and descent measures were analyzed separately using SPSS (ver.24, IBM). For handrail use, whole body orientation and stepping strategy, we ran Chi-Squared tests, with age-group and going as independent variables. For the other measures, a mixed linear model was used. Age group (2 levels: young, old), going (4 levels: 175, 225, 275, and 325 mm) and start-condition (2 levels: standing, walking) were fixed factors, whereas participant was the random factor. The three-way interactions are not reported here. Least significant difference (LSD) *post-hoc* test was used to investigate significant effects. For the single step clearance differences between age groups for each direction (ascent/descent) and start condition (standing/walking start) were assessed using an ANOVA test.

To compare between groups, left and right leg and foot lengths were averaged for each participant. The group mean and SE for height, leg and foot lengths were calculated. Additionally, the mean and SE were calculated for the data collected on the second visit (balance and questionnaires). The difference between younger and older participants in these quantities was then assessed using a *t*-test.

In order to determine the factors affecting stair performance, regression analyses were run for the younger and older group separately. The analyses were run for foot clearance in ascent, and foot overhang in descent. The factors included in the analyses were chosen to explain the possible influence on stair gait performance. For this reason, balance ability (Svanstrom, 1974; Tinetti et al., 1988; Lord et al., 2007) and hours of physical activity per week (proxy for physical ability) (Svanstrom, 1974; Tinetti et al., 1988; Lord et al., 2007) were used in ascent and descent. Additional factors, such as medications taken (Tinetti et al., 1988; Lord et al., 2007) and fear of falling (Lord et al., 2007) have been shown to be related to performance in stair descent and were included in the analysis. Additionally, mean foot length was included for stair descent to account for differences in anthropometric dimensions, which seem more relevant for foot placement in stair descent.

## Results

### Stair Negotiation Performance

#### Ascent

**Figure 2:**
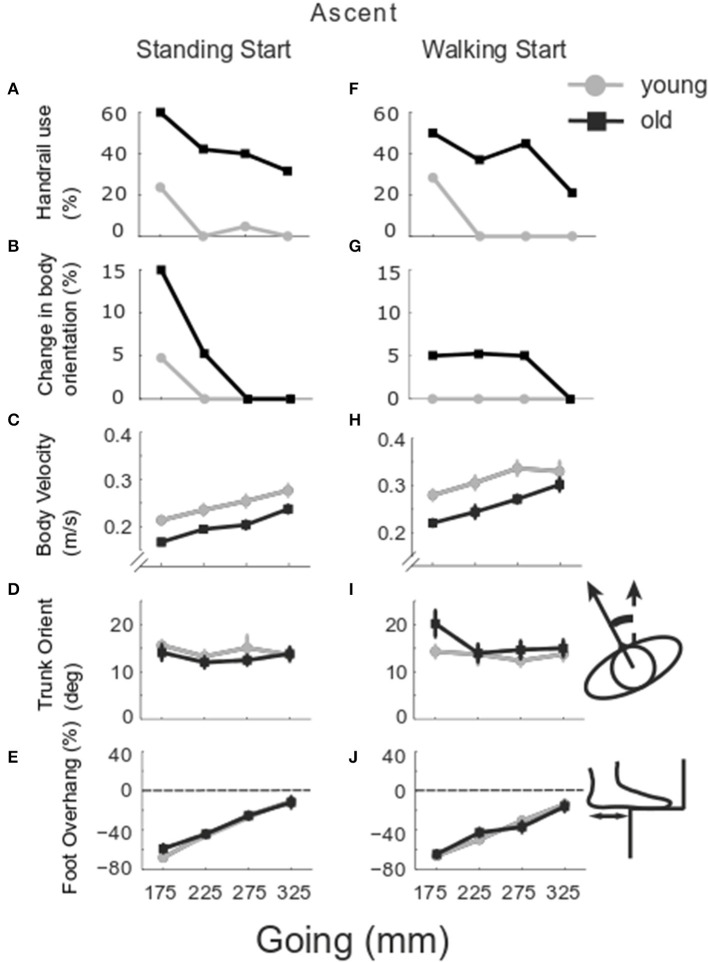
Group stair performance in ascent. The percentage of trials in which the handrail was used **(A,F)** and the whole body turned toward one handrail are reported **(B,G)** for older (black) and younger group (gray). The group mean and SE of body velocity **(C,H)**, trunk orientation **(D,I)** and foot overhang relative to foot length **(E,J)** are reported for the goings investigated −175, 225, 275, and 325 mm from standing (left column) and walking start (right column). Trunk orientation was calculated relative to the trunk position whilst standing at the beginning of the recording and used as the baseline (0deg here). Foot overhang is reported as % of antero-posterior foot length, 0% means that the whole foot is placed on the step, negative values show the percentage of foot outside the step at foot landing.

At any given going, older participants used the handrail more often than younger participants (Figures 2A,F) in both standing start trials [*Group* Pearson χ^2^: Going175 χ^2^(df = 1, *N* = 41)=5.528, *p* = 0.019; G225 χ^2^(1,40) = 11.053, *p* = 0.001; G275 χ^2^(1,41) = 7.424, *p* = 0.006; G325 χ^2^(1,40) = 7.802, *p* = 0.005] and walking start trials, except for 175 mm-going [*Group* G175 χ^2^(df = 1, *N* = 41) = 1.977, *p* = 0.160; G225 χ^2^(1,40) = 9.378, *p* = 0.002; G275 χ^2^(1,41) = 12.108, *p* = 0.001; G325 χ^2^(1,39) = 4.692, *p* = 0.030]. The younger group used the handrail mainly for the smallest going [*Going* standing-start χ^2^ (df = 3, *N* = 84) = 12.205, *p* = 0.007; walking-start χ^2^ (3,83) = 19.095, *p* < 0.001]. Body orientation was not affected by group or going (Figures 2B,G). Participants always negotiated the steps with alternate feet.

Gait velocity was slower in older participants (*Group p* < 0.001), from a standing start (*Start p* < 0.001), and for smaller goings (*Going p* = 0.033) (Figures 2C,H). Trunk orientation (Figures 2D,I) changed with start condition (*Start p* = 0.045) and the older group rotated the trunk more in the walking start trials (*Group x Start p* = 0.011). Foot overhang (Figures 2E,J) increased with smaller goings (*Going p* < 0.001).

**Figure 3:**
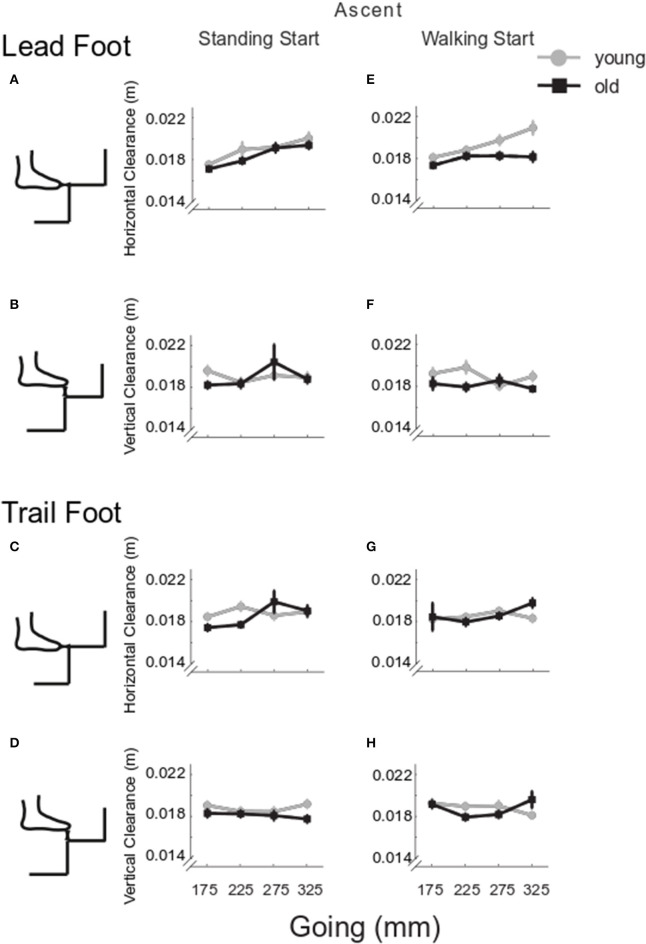
Group clearance in ascent. The group mean and SE for older (black) and younger (gray) clearance in stair ascent was calculated in the horizontal direction (direction of travel) and in the vertical direction for lead and trail foot, at the four goings. The mean values were calculated whilst negotiating the stair from a standing start (left column) and from a walking start (right column). Lead foot clearance in horizontal **(A,E)** and vertical direction **(B,F)**; trail foot clearance in horizontal **(C,G)** and vertical direction **(D,H)**.

**Figure 4:**
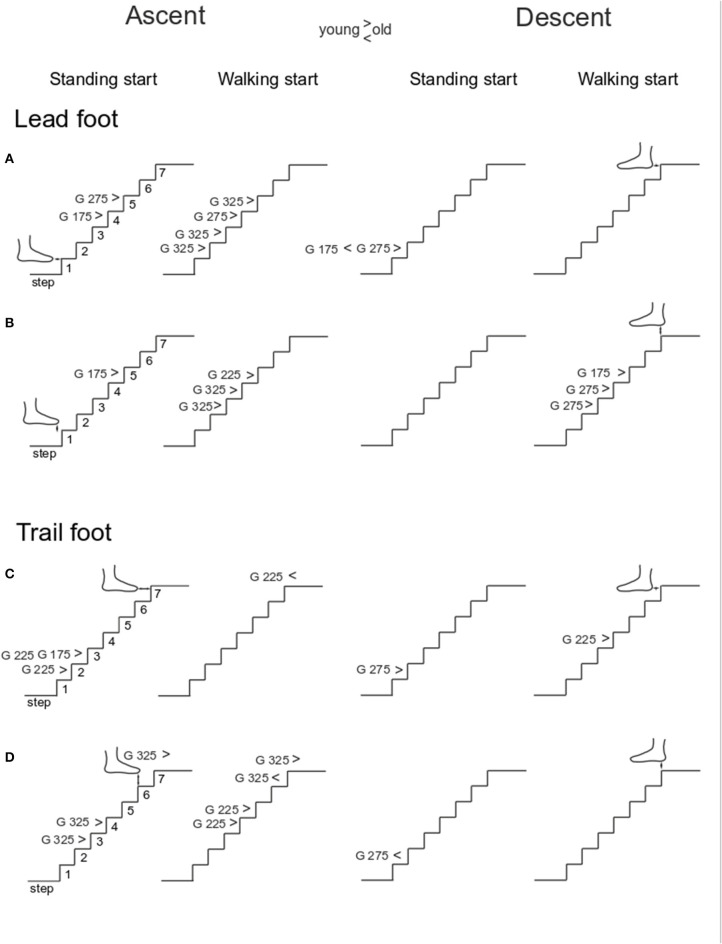
Single step clearance. The group average of foot clearances was calculated for each step and an ANOVA test was run to find differences between younger and older groups for each step in each stair configuration. The results reported here are divided in four columns: ascent standing start, ascent walking start, descent standing start and descent walking start. For each panel, the significant difference (*p* < 0.05) is reported with the indication of which going (G) showed the significant results and whether the younger group's means was higher (>) or lower than older group's mean (<). The four rows show: Lead foot clearance in horizontal **(A)** and vertical direction **(B)**; trail foot clearance in horizontal **(C)** and vertical direction **(D)**.

Lead foot horizontal clearance (Figures 3A,E) was smaller in the older group (*Group p* < 0.001) and decreased as going decreased (*Going p* = 0.001). The older group showed a larger clearance in standing start trials (*Group x Start p* = 0.046). Lead vertical clearance (Figures 3B,F) was smaller in the older group for the smaller goings (*Going x Group p* = 0.034). Trail foot horizontal clearance was smaller in the older group for the smaller goings (*Going x Group p* = 0.017) (Figures 3C,G), and the vertical clearance showed a significant *Going x Start* interaction (*p* = 0.006) indicating that the smaller goings differed between the two start conditions (G175 *p* = 0.035; G225 *p* = 0.002; G275 *p* = 0.003) (Figures 3D,H). Examining all the configurations for both standing and walking start, when a significant difference in clearance was found at a step, usually the clearance in the younger group was greater than the older group. The central steps (2–4) showed the majority of occurrences of significant differences between the younger and older groups (Figure 4, left columns).

**Figure 5:**
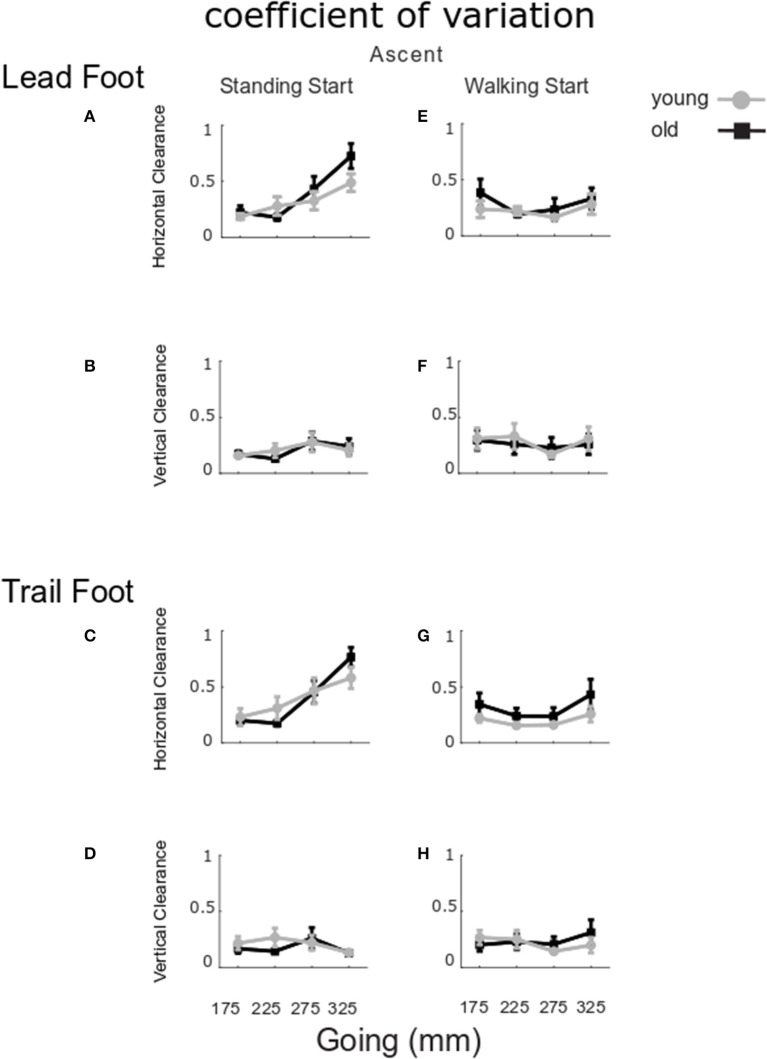
Coefficient of variation for clearance in ascent. The group mean and SE for older (black) and younger (gray) coefficient of variation for clearance in stair ascent at the four goings. The values were calculated for standing start (left column) and walking start (right column). Coefficient of variation for lead foot clearance in horizontal **(A,E)** and vertical direction **(B,F)**; trail foot clearance in horizontal **(C,G)** and vertical direction **(D,H)**.

The coefficient of variation in foot clearances is shown in Figure 5. No significant group differences for horizontal lead foot clearance could be found (Figures 5A,E, *Group p* = 0.219). However, the coefficient of variation was larger for the standing start condition (*Start p* = 0.008). In addition, a going effect was found (*Going p* < 0.001) indicating that the 325 mm-going induced a higher coefficient of variation then all the other goings. A *Going x Start* interaction was also found (*p* = 0.001) and *post-hoc* analysis showed that the 325 mm-going induced a higher variation only for the standing start trials. Similar results were found for the horizontal clearance coefficient of variation for the trail leg (Figures 5B,F) as clearance variation was larger for the standing start condition (*Start p* < 0.001), and for the largest going G325 (*Going p* < 0.001), but there was no significant group effect (*Group p* = 0.284). A significant *Going x Start* interaction (*p* = 0.001) indicated that the 325 mm-going induced a higher variation only for the standing start trials.

For the vertical clearances (Figures 5C,D,G,H), the coefficient of variation did not show any significant group, start condition or going effects for the lead foot (*Group p* = 0.950, *Start p* = 0.078, *Going p* = 0.952) or the trail foot (*Group p* = 0.951, *Start p* = 0.230, *Going p* = 0.887).

**Figure 6:**
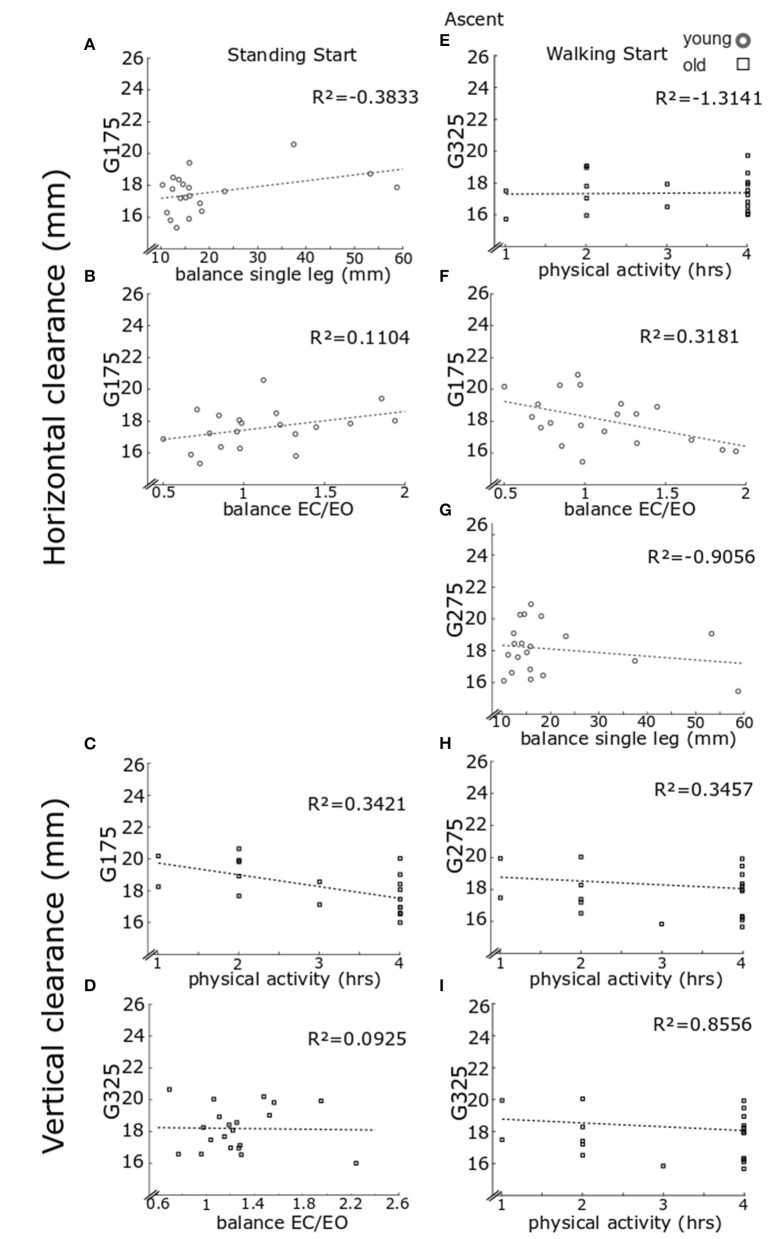
Regression model for clearance in ascent. Lead foot clearance regression model results. Each panel reports individual data for older (black) and younger (gray) clearance in stair ascent in the horizontal direction and in the vertical direction. As only the lead foot showed significant results from the regression models, only these data are reported. Results are reported for standing start (left column) and walking start (right column). Standing start. Lead foot clearance in the horizontal direction for going of 175 mm for younger participants relative to balance score on one leg **(A)** and balance eyes closed over eyes open **(B)**. Lead foot vertical clearance for going of 175 mm for older participants relative to hours of physical activity **(C)** and for going of 325 mm relative to balance eyes closed over eyes open **(D)**. Walking start. Lead foot clearance in the horizontal direction for going of 325 mm for older participants relative to hours of physical activity **(E)**. Clearance for going of 175 mm for younger participants relative to balance eyes closed over eyes open **(F)** and at going of 275 mm relative to balance score on one leg **(G)**. Lead foot vertical clearance for older participants relative to hours of physical activity for going of 275 mm **(H)** and for going of 275 mm **(I)**. For each panel a least square fit line and the *R*^2^ values are reported.

The regression analyses (Figure 6) for the older participants, showed that vertical clearance in the standing start trials at a going of 175 mm was lower for participants who reported a higher number of hours of physical activity [Figure 6C, *F*_(1, 18)_ = 9.3613, *p* = 0.0067, *R*^2^ = 0.3421, correlation coefficient *b* = −0.7399], whilst at a going of 325 mm, the clearance was higher for participants who had a higher score in balance EC vs. EO [Figure 6D, *F*_(1, 17)_ = 10.2991, *p* = 0.0051, *R*^2^ = 0.0925, *b* = 2.8092]. Vertical clearance in the walking start trials was lower for the participants reporting more hours of physical activity per week at a going of 275 mm [Figure 6H, *F*_(1, 18)_ = 4.4934, *p* = 0.0482, *R*^2^ = 0.3457, *b* = −0.9418] or 325 mm [Figure 6I, *F*_(1, 17)_ = 5.3088, *p* = 0.0341, *R*^2^ = 0.8556, *b* = −0.4810]. Horizontal clearance in walking start trials was lower for participants reporting fewer hours of physical activity at a going of 325 mm [Figure 6E, *F*_(1, 17)_ = 5.5676, *p* = 0.0305, *R*^2^= −1.3141, *b* = −0.8736].

For the younger group, the step-wise regression model showed that the horizontal foot clearance in standing start trials at a going of 175 mm [model *F*_(2, 15)_ = 6.7106, *p* = 0.0083] increased for participants with larger the single-leg balance score (Figure 6A, step-1 of the regression *b* = 0.0760, *p* = 0.0066, *R*^2^= −0.3833), or larger balance EC vs. EO score (Figure 6B, step-2 of the regression *b* = 1.7499, *p* = 0.0193, *R*^2^ = 0.1104). Horizontal clearance in walking start trials was greater for participants with a larger balance EC vs. EO score at a going of 175 mm [Figure 6F, *F*_(1, 16)_ = 5.0875, *p* = 0.0385, *R*^2^ = 0.3181, *b* = −1.8319), or larger single-leg balance score at a going of 275 mm [Figure 6G, *F*_(1, 16)_ = 5.2194, *p* = 0.0363, *R*^2^ = −0.9008, *b* = −0.0848).

#### Descent

**Figure 7:**
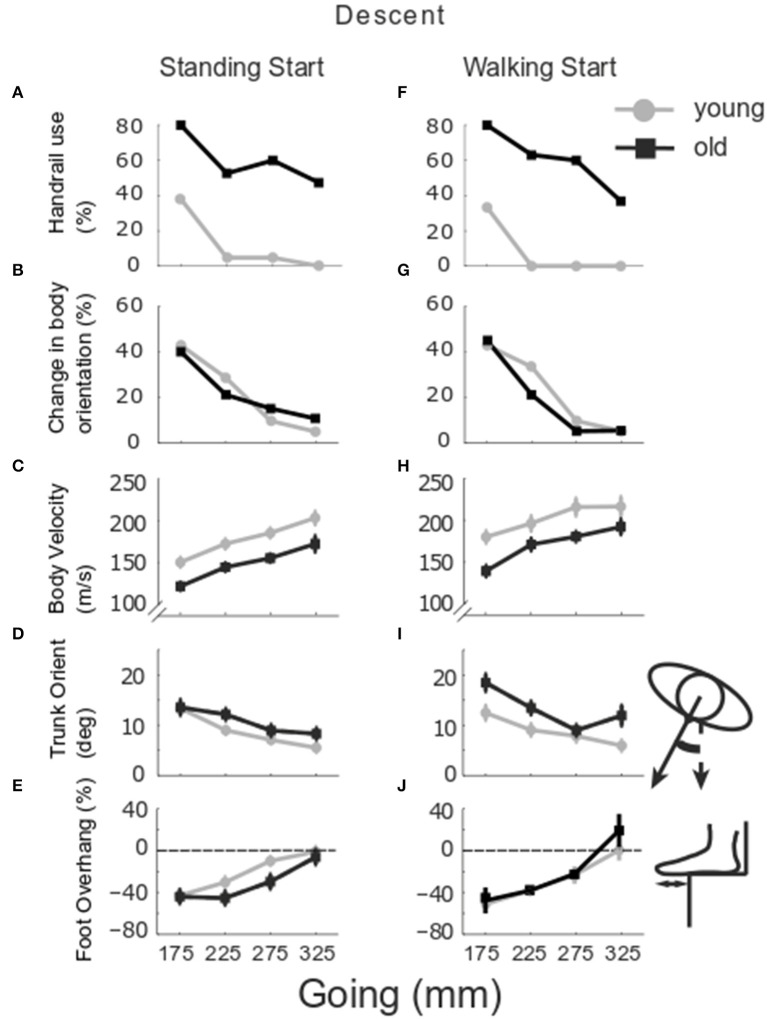
Group stair performance in descent. The percentage of trials in which the handrail was used **(A,F)** and the whole body turned toward one handrail is reported **(B,G)** for older (black) and younger group (gray). Group mean and SE of body velocity **(C,H)**, trunk orientation **(D,I)** and foot overhang **(E,J)** relative to stair going, in standing (left column) and walking start trials (right column).

At any given going, the older group used the handrail more often than younger participants (Figures 7A,F) in both standing start trials [*Group* G175 χ^2^ (df = 1, *N* = 41) = 7.411, *p* = 0.006; G225 χ^2^(1,40) = 11.465, *p* = 0.001; G275 χ^2^(1,41) = 14.435, *p* < 0.001; G325 χ^2^(1,40) = 12.835, *p* < 0.001] and walking start trials [*Group* G175 χ^2^(df = 1, *N* = 41) = 9.058, *p* = 0.003; G225 χ^2^(1,40) = 18.947, *p* < 0.001; G275 χ^2^(1,41) = 18.814, *p* < 0.001; G325 χ^2^(1,39) = 8.980, *p* = 0.003]. The younger group used the handrail more often for the smallest goings, especially for G175 [*Going* standing-start χ^2^(df = 3, *N* = 84) = 18.616, *p* < 0.001; walking-start χ^2^(3,83) = 22.570, *p* < 0.001]. Whole body orientation (Figures 7B,G) was affected by going for the younger group for both start conditions [*Going* standing-start χ^2^(df = 3, *N* = 84) = 11.596, *p* = 0.009; walking-start χ^2^(3,83) = 11.793, *p* = 0.008], whilst for the older group, it was only affected in the walking start trials [*Going* χ^2^(df = 3, *N* = 78) = 13.585, *p* = 0.004]. At a going of 175 mm, the older group placed both feet on the same step in 10 and 5% of the standing and walking start trials, respectively, and in 5.3% of the standing start trials at a going of 325 mm. No relationship between change in stepping strategy and age group or going was found.

**Figure 8:**
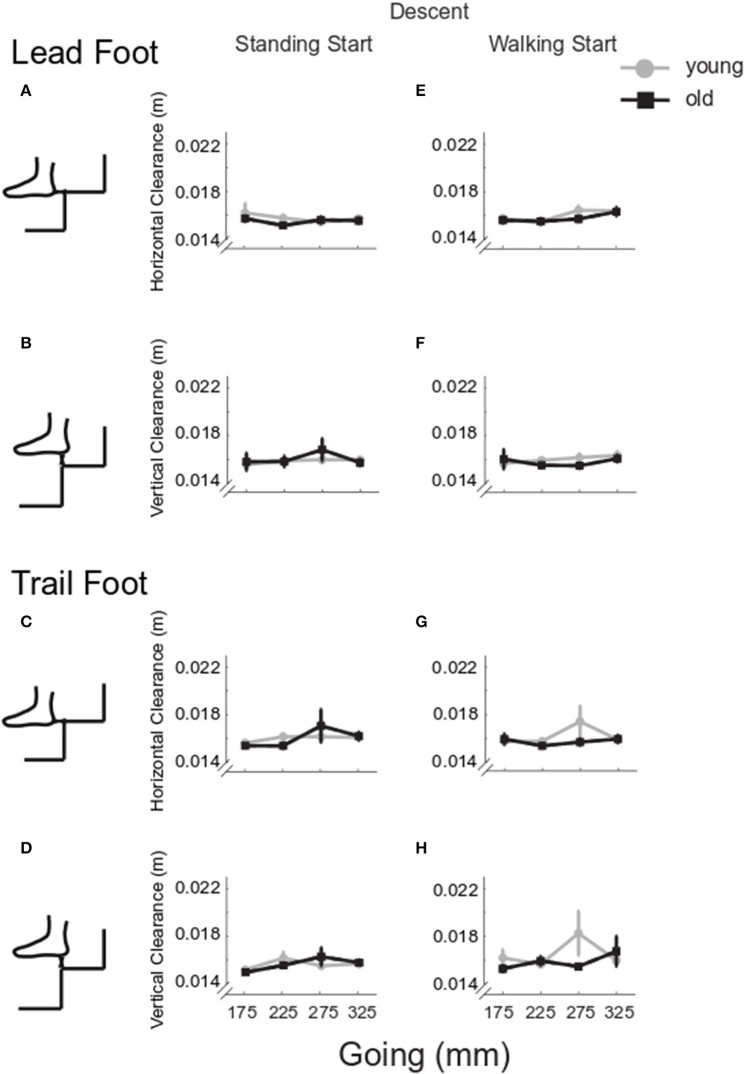
Group clearance in descent. Group mean and SE for older (black) and younger (gray) lead and trail foot horizontal **(A,C,E,G)** and vertical clearance **(B,D,F,H)** in stair descent at the four goings, from a standing (left column) and a walking start (right column).

Figures 7C,H show that body velocity was lower in older participants (*Group p* = 0.001), from a standing start (*Start p* = 0.001) and for smaller goings (*Going p* = 0.003). Change in trunk orientation (Figures 7D,I) was larger in older participants (*Group p* = 0.017) and increased in both groups as going decreased (*Going p* = 0.002). The older group rotated the trunk more in walking start trials (*Group x Start p* = 0.015). Foot overhang was larger for the smaller goings (*Going p* < 0.001) (Figures 7E,J). For the trail foot (Figures 8C,D,G,H), an interaction was found for the vertical clearance (*Going x Start p* = 0.039) indicating that only the 275 mm-going was different between the two start conditions. No significant differences were found for the lead foot (Figures 8A,B,E,F). Examining the single step for all the configurations for both standing and walking task (Figure 4, right columns), step 1 (at the end of stair negotiation in descent) showed the majority of occurrences of differences between younger and older groups with two instances in which the clearance in the older group was higher than the younger group (horizontal lead foot stand start at 175 mm *p* = 0.001, and vertical trail foot stand start at 275 mm *p* = 0.028).

**Figure 9:**
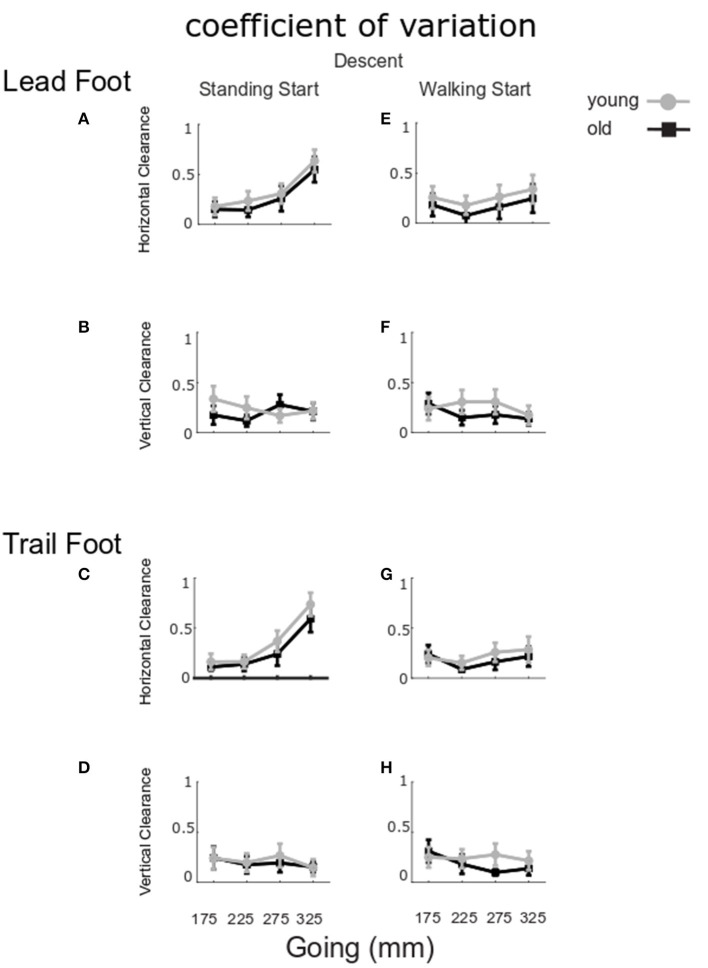
Coefficient of variation for clearance in descent. The group mean and SE for older (black) and younger (gray) of the coefficient of variation for lead and trail foot horizontal **(A,C,E,G)** and vertical clearance **(B,D,F,H)** in stair descent at the four goings, from a standing (left column) and a walking start (right column).

There was no statistically significant difference in coefficient of variation for horizontal lead foot clearance (Figures 9A,E, *Group p* = 0.400). However, the coefficient of variation was larger for standing start trials (*Start p* = 0.034). In addition, a going effect was found (*Going p* < 0.001) indicating that the 325 mm-going induced a higher coefficient of variation then all the other goings. A significant *Going x Start* interaction (*p* = 0.045) indicated that 325 mm-going induced a higher variation only for the standing start trials. Similar results were found for the horizontal trail leg (Figures 9B,F), as variation was larger for the standing start condition (*Start p* = 0.004), and for the largest going G325 (*Going p* < 0.001), but no differences between groups could be found (*Group p* = 0.429). A significant *Going x Start* interaction (*p* < 0.001) indicated that the 325 mm-going induced a higher variation only for the standing start trials.

For the vertical clearances (Figures 9C,D,G,H), the coefficient of variation did not show any significant group, start condition or going effects for the lead foot (*Group p* = 0.523, *Start p* = 0.948, *Going p* = 0.698) or the trail foot (*Group p* = 0.681, *Start p* = 0.804, *Going p* = 0.446).

**Figure 10:**
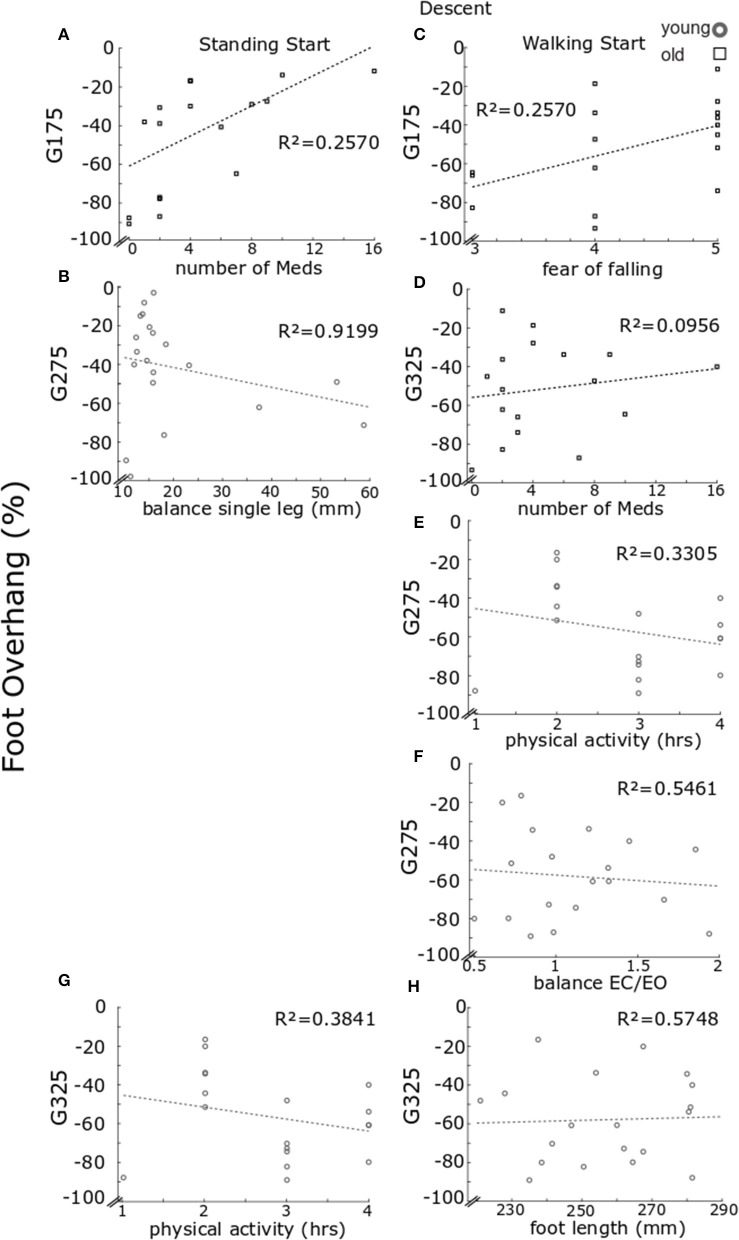
Regression model results for foot overhang in descent. Regression model results for foot overhang. Each panel reports individual data for older (black) and younger (gray) participants. Results are reported for standing start **(A–B)** and walking start **(C–H)**. Standing start. Overhang for going of 175 mm for older participants relative to number of medications taken **(A)** and for younger participants at goings of 275 mm relative to balance on one leg **(B)**. Walking start. Foot overhang for older participants at going of 175 mm relative fear of falling score **(C)** and at going of 325 mm relative to number of medications taken **(D)**. For the younger individuals, foot overhang at going of 275 mm relative to hours of physical activity **(E)** and balance eyes closed over eyes open **(F)**; while at going of 325 mm relative to hours of physical activity **(G)** and foot length **(H)**. For each panel a least square fit line and the *R*^2^ values are reported.

The regression analyses (Figure 10) showed that for the older participants, foot overhang in standing start trials was larger for participants who reported taking fewer medications at a going of 175 mm [Figure 10A, *F*_(1, 16)_ = 6.6058, *p* = 0.0205, *R*^2^ = 0.2922, *b* = 3.8815] and at going of 325 mm in walking trials [Figure 10D, *F*_(1, 15)_ = 8.3254, *p* = 0.0113, *R*^2^ = 0.0956, *b* = 4.2408]. In walking start trials foot overhang was larger for participants reporting a lower score for fear of falling at a going of 175 mm [Figure 10C, *F*_(1, 15)_ = 5.1884, *p* = 0.0378, *R*^2^ = 0.2570, *b* = 15.8498].

For the younger group, foot overhang in standing start trials at a going of 275 mm was larger for participants with a higher score in the single-leg balance test [Figure 10B, *F*_(1, 15)_ = 24.6760, *p* < 0.001, *b* = −0.8750, *R*^2^ = 0.9199] and, in walking start trials [*F*_(2, 14)_ = 7.3830, *p* = 0.0065] for participants reporting higher hours of physical activity (Figure 10E, step-1 of the regression *b* = −11.0459, *p* = 0.0128, *R*^2^ = 0.3305) or a higher score in the balance EC vs. EO test (Figure 10F, step-2 of the regression *b* = −26.8983, *p* = 0.0199, *R*^2^ = 0.5461). Foot overhang at a going of 325 mm in walking trials [*F*_(2, 13)_ = 9.6329, *p* = 0.0027) was predicted by hours of physical activity with larger overhang for higher number of hours (Figure 10G, step-1 of the regression *b* = −15.2187, *p* = 0.0017, *R*^2^ = 0.3841) or smaller foot length (Figure 10H, step-2 of the regression *b* = −4.6799, *p* = 0.0276, *R*^2^= 0.5748).

### Functional Capability Assessments

Although older participants' height was lower than the younger group (mean ± standard error “SE” younger 1.75 ± 0.003 m, older 1.66 ± 0.003 m, *p* = 0.0206), no statistical difference between groups was found for leg length (younger 0.854 ± 0.017 m, older 0.833 ± 0.016 m, *p* = 0.3745) or foot length (younger 0.259 ± 0.045 m, older 0.251 ± 0.041 m, *p* = 0.2317).

**Figure 11:**
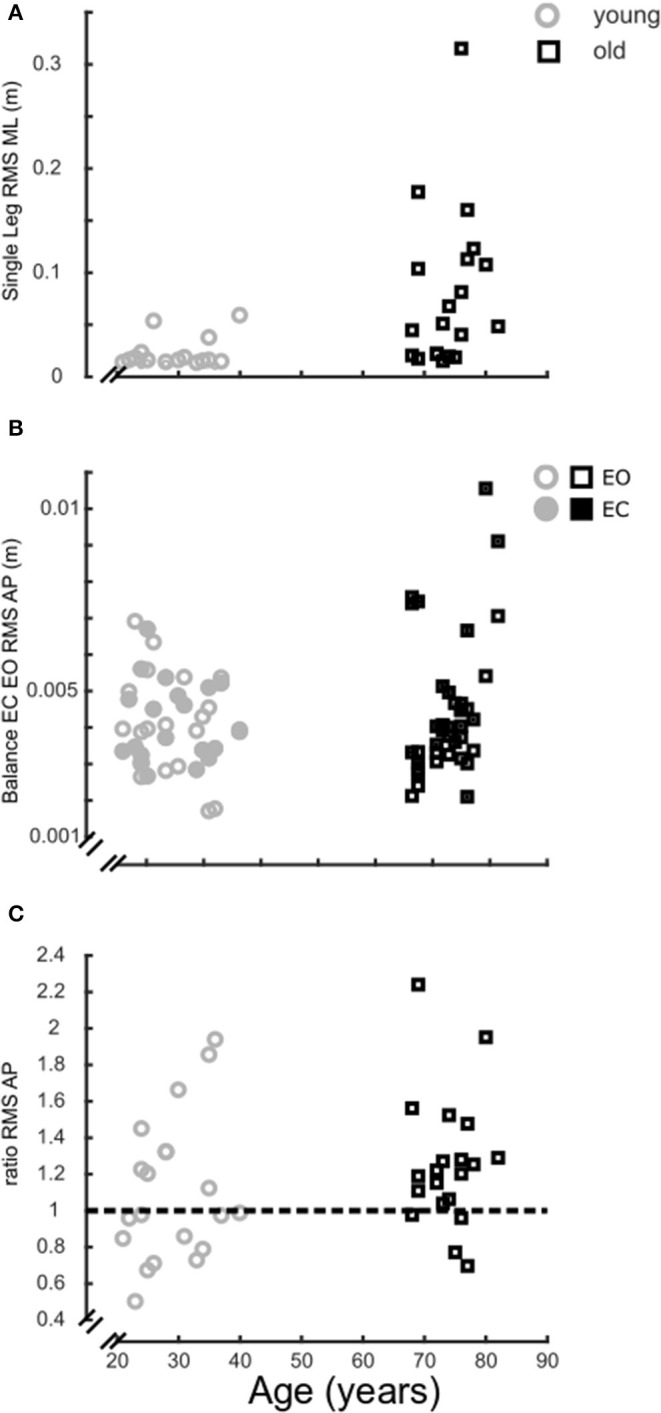
Balance ability. Balance performance over age, grouped in older (black) and younger participants (gray). **(A)**, Medio-lateral root mean square (RMS) of the CoP trace of the single leg balance trial (5 s); **(B)**, Antero-posterior RMS of the CoP trace for the quiet standing trials (30 s) with eyes closed (filled) and eyes open (open); **(C)**, Ratio of the antero-posterior RMS CoP for the quiet standing trials with eyes closed over eyes open trials.

*Single-leg balance*. The medio-lateral RMS of CoP was greater in the older group than the young group (older 0.079 ± 0.017 m, younger 0.020 ± 0.003 m, *p* < 0.001) (Figure 11A).

*Balance EC vs. EO*. With EC, the antero-posterior RMS of CoP was greater in the older group (older 0.005 ± 0.0005 m, younger 0.0041 ± 0.0002 m, *p* = 0.035). There was no difference between age groups with EO (older 0.004 ± 0.0003 m, younger 0.0041 ± 0.0003 m, *p* = 0.907) (Figure 11B) and for the ratio of RMS CoP between EC and EO (older 1.26 ± 0.08, younger 1.10 ± 0.09, *p* = 0.398) (Figure 11C).

*Questionnaires*. Weekly hours of physical activity were similar between groups (older 3.1 ± 0.3 h, younger 2.7 ± 0.2 h, *p* = 0.2913). The older group reported a higher fear of falling (older 0.7 ± 0.2, younger 0.2 ± 0.1, *p* = 0.0185) and a higher number of medications taken (older 4.4 ± 0.9, younger 0.2 ± 0.1, *p* < 0.001).

## Discussion

Older and younger participants negotiated an experimental stair with different step going sizes and two different methods of approaching the flight of stairs. Here we discuss the age differences in stair performance in response to going manipulation and start condition.

### Differences Between Younger and Older Participants on Stairs: Effect of Aging

Older participants used the handrail more often, had a lower gait velocity and smaller foot clearances. These results are unlikely to be related to the participants' anthropometry because the only group difference was a small difference in height, with no difference in leg or foot length. However, the adaptations shown by the older group may be underpinned by an aging-induced deterioration of musculoskeletal capabilities (Prince et al., 1997; Menz et al., 2003b; Iosa et al., 2014). In ascent, demands on the musculoskeletal system are heightened, because the body mass is moved against gravity. Using the handrail helps in propelling the body upwards. Considering that hours of physical activity was a predictor of older participants' clearance in ascent, it is likely that older participants used the handrail more to compensate for their real or perceived reduced muscle strength. In fact, counterintuitively, the participants that reported a higher level of physical activity showed lower clearances and higher overhang suggesting an increase in confidence or a more tuned strategy, but potentially closer to the risk level. As older individuals tend to employ a higher joint moment on stairs relative to their maximum (Reeves et al., 2008, 2009) compared to younger individuals, it is possible that a higher fitness level is related to a more efficient strategy, without using excessive energy to perform the task successfully.

Older participants also used the handrail more often in descent. The muscle strength demands of descent are lower because no work against gravity is needed, and eccentric muscle strength is better preserved in older people (Roig et al., 2010). However, using the handrails helps with stability, particularly in the single support phase. The need for additional support is consistent with the predictors of older participants' performance in descent which are related to their confidence and overall health level (fear of falling and medications). Consistent with older participants' lower physical abilities and confidence, the older group self-selected stair walking speed was slower, probably to use more time to perform accurate stepping and better cope with the demands of the task, allowing them to produce larger joint forces (relative to their maximum capability) at slower velocities.

Older participants successfully adapted to the changes in environment without accidents. Indeed, the change in going size affected both groups similarly. The smallest going tested here, 175 mm, appeared to be challenging for both groups, as also the younger individuals occasionally used the handrail in both ascent and descent. This may seem at odds with the finding that older people had larger medio-lateral sway in the single leg balance test, particularly as stair negotiation involves periods when the body center of mass is either being lowered or elevated during single stance. These results add to the debate on the relevance of static balance tests as useful predictors of fall risk, as there is no general consensus on the relationship between static balance tests and real-life fall risk (Menz et al., 2003a; Lord et al., 2007; Granata and Lockhart, 2008).

An indication that the system was close to a risk-threshold was displayed by the older group's smaller foot clearance, particularly at smaller goings (Going x Group interaction for horizontal trail foot and vertical lead foot clearances). Changes in step going alter the demands of the task, and may increase the likelihood of accidents and injury (Roys, 2001; Novak et al., 2016), particularly if users are less able to accurately assess these demands, which is a problem for older individuals due to the slowly progressing (rather than acute) decline in neuro-musculo-skeletal capabilities. Although the older group's clearance at 175 mm-going indicated a heightened risk, we showed that this going was the most challenging one for both groups. Unexpectedly, we did not find a reliable effect of the 225 mm or the 325 mm goings. The 225 mm-going is just within the UK guidelines for private buildings and does not allow complete foot placement, on average. For this reason, we expected a potential effect of this going, but our tests only showed a quasi-linear trend for most of the quantities measured, with stair gait becoming less affected as the going was larger. On the other hand, a potential drop in locomotor performance at the largest going (325 mm) could be expected because this going may impose a larger than comfortable stride. However, in this study we showed that 325 mm-going did not seem to worsen performance, but only increased variability in the task as shown by the higher coefficient of variation for horizontal clearance. More work is needed to determine the precise relationship between going size and participants' anthropometry and the role of an increased variability in efficient motor control. It is also noteworthy that in order to identify reliable predictors of stair falls, precise quantification of performance on the stairs and its relationship with other tests should be investigated, as static standing tests were not able to account for the differences in stair performance tested here. This is needed to investigate the mechanisms of accidents to improve falls prevention, particularly in older people.

### Older Group's Increased Difficulty With Walking Start Condition

Here standing and walking start were compared to investigate two different modes of initiation and termination of locomotion, the effect of disrupting the rhythmicity of the task, and the ability to react to changes in environment (Wollacott and Tang, 1977; Menz et al., 2003a). Older participants seemed to experience more difficulties with a walking start (Group × Start interactions); in fact, they rotated their trunk more and had a smaller horizontal lead foot clearance. In this experiment, the transition in tasks could be planned because participants were free to see the staircase before negotiating it and the visual input was not manipulated in any way. For this reason, the older group's adaptations are unlikely to be related to a reduced reaction time in this group. However, with the standing start condition, the initiation of movement requires a process of adjustments and anticipatory reactions and control (Patla, 2003) that destabilizes the system in order to allow movement. This is reflected in the higher coefficient of variation for clearance in ascent and descent. On the other hand, in the walking start condition, a transition between two locomotor processes is needed. This would predict a more optimal strategy in walking start trials. However, the motor tuning necessary to change the motor task may be difficult for older participants, particularly considering the higher body velocity and consequent higher momentum, both of which require control during stair negotiation, but especially in the single support phase in stair descent. This extra level of control may increase the complexity of the overall control in walking start trials, which could explain the increased difficulty for older participants in this study. Therefore, pausing before negotiating a staircase after level walking (either before or in between flights of stairs on a level landing) may make stair walking safer by allowing more time to assess the environment, plan the motor task, and subsequently execute it in a less risky body posture and at lower momentum.

In conclusion, in this study we have shown the difference in stair gait according to going dimension in younger and older individuals. We have found that smaller goings induced significant adaptations in both groups, and healthy older participants showed motor adaptations and strategies consistent with increased difficulty, compared to the younger cohort. We also found that older participants showed additional difficulties when stair negotiation was preceded by level gait, as a transition in motor control was required in the two tasks. This suggests that stair design should allow comfortable gait for everyone, and in particular for older individuals, and that pausing before negotiating a staircase could be a safer strategy.

## Data Availability Statement

The datasets generated for this study are available on request to the corresponding author.

## Ethics Statement

The studies involving human participants were reviewed and approved by Institute for Biomedical Research into Human Movement and Health, Manchester Metropolitan University. The patients/participants provided their written informed consent to participate in this study.

## Author Contributions

The experiments were performed at the Laboratory of Biomechanics, School of Healthcare Science, Manchester Metropolitan University, Manchester, UK. ID, NR, VB, and CM contributed to the conception of the experiment, data acquisition, analysis, and interpretation. ID wrote the article. All authors contributed to design of the work, contributed to the critical review of the manuscript, and approved the final version.

## Conflict of Interest

MR was self-employed as a consultant in the company Rise and Going Consultancy. The remaining authors declare that the research was conducted in the absence of any commercial or financial relationships that could be construed as a potential conflict of interest.

## References

[B1] Age U. K. (2012). Stop Falling: Start Saving Lives and Money. London. Available online at: https://www.ageuk.org.uk/globalassets/age-uk/documents/reports-and-publications/reports-and-briefings/health--wellbeing/rb_oct11_stop_falling_report.pdf

[B2] Begg R. K. Sparrow W. A. (2000). Gait characteristics of young and older individuals negotiating a raised surface: implications for the prevention of falls. J. Gerontol. A Biol. Sci. Med. Sci. 55A, M147–M154. 10.1093/gerona/55.3.M147 10795727

[B3] Cavanagh P. R. Mulfinger L. M. Owens D. A. (1997). How do the elderly negotiate stairs. Muscle Nerve Suppl. 5, S52–55. 10.1002/(SICI)1097-4598(1997)5+<52::AID-MUS13>3.0.CO;2-0 9331385

[B4] Government. H. M (2010). Protection From Falling, Collision and Impact. Approved Document, K in Building Regulations 2010, Draft Edition 2013 (Geneva).

[B5] Granata K. P. Lockhart T. E. (2008). Dynamic stability differences in fall-prone and healthy adults. J. Electromyogr. Kinesiol. 18, 172–178. 10.1016/j.jelekin.2007.06.008 17686633 PMC2895268

[B6] Iosa M. Fusco A. Morone G. Paolucci S. (2014). Development and decline of upright gait stability. Front. Aging Neurosci. 6:14. 10.3389/fnagi.2014.00014 24550829 PMC3913994

[B7] Jacobs J. V. (2016). A review of stairway falls and stair negotiation: lessons learned and future needs to reduce injury. Gait Posture 49, 159–167. 10.1016/j.gaitpost.2016.06.030 27427833

[B8] Jahn K. Zwergal A. Schniepp R. (2010). Gait disturbances in old age. Dtsch. Arztebl. Int. 107, 306–316. 10.3238/arztebl.2010.0306 20490346 PMC2872829

[B9] Lord S. R. Lloyd D. G. Li S. K. (1996). Sensori-motor function, gait patterns and falls in community-dwelling women. Age Ageing 25, 292–299. 10.1093/ageing/25.4.292 8831874

[B10] Lord S. R. Sherrington C. Menz H. B. Close J. C. T. (2007). Falls in Older People. 2nd ed. (Cambridge: Cambridge University Press), 26–167. 10.1017/CBO9780511722233

[B11] McFadyen B. J. Winter D. A. (1998). An integrated biomechanical analysis of normal stair ascent and descent. J. Biomech. 21, 733–744. 10.1016/0021-9290(88)90282-5 3182877

[B12] Menz H. B. Lord S. R. Fitzpatrick R. C. (2003a). Acceleration patterns of the head and pelvis when walking on level and irregular surfaces. Gait Posture 18, 35–46. 10.1016/S0966-6362(02)00159-5 12855299

[B13] Menz H. B. Lord S. R. Fitzpatrick R. C. (2003b). Age-related differences in walking stability. Age Ageing 32, 137–142. 10.1093/ageing/32.2.137 12615555

[B14] Novak A. C. Komisar V. Maki B. E. Fernie G. R. (2016). Age-related differences in dynamic balance control during stair descent and effect of varying step geometry. Appl. Ergon. 52, 275–284. 10.1016/j.apergo.2015.07.027 26360219

[B15] Patla A. E. (2003). Strategies for dynamic stability during adaptive human locomotion. IEEE Eng. Med. Biol. Mag. 22, 48–52. 10.1109/MEMB.2003.1195695 12733458

[B16] Prince F. Corriveau H. Hebert R. Winter D. A. (1997). Gait in the elderly. Gait Posture 5, 128–135. 10.1016/S0966-6362(97)01118-1

[B17] Reeves N. D. Spanjaard M. Mohagheghi A. A. Baltzopoulos V. Maganaris C. N. (2008). The demands of stair descent relative to maximum capacities in elderly and young adults. J. Electromyogr. Kinesiol. 18, 218–227. 10.1016/j.jelekin.2007.06.003 17822923

[B18] Reeves N. D. Spanjaard M. Mohagheghi A. A. Baltzopoulos V. Maganaris C. N. (2009). Older adults employ alternative strategies to operate within their maximum capabilities when ascending stairs. J. Electromyogr. Kinesiol. 19, e57–e68. 10.1016/j.jelekin.2007.09.009 18053743

[B19] Riener R. Rabuffetti M. Frigo C. (2002). Stair ascent and descent at different inclinations. Gait Posture 15, 32–44. 10.1016/S0966-6362(01)00162-X 11809579

[B20] Rogers M. W. Hedman L. D. Johnson M. E. Martinez K. M. Mille M.-L. (2003). Triggering of protective stepping for the control of human balance: age and contextual dependence. Brain Res. Cogn. Brain Res. 16, 192–198. 10.1016/S0926-6410(02)00273-2 12668227

[B21] Roig M. MacIntyre D. L. Eng J. J. Narici M. V. Maganaris C. N. Reid W. D. (2010). Preservation of eccentric strength in older adults: evidence, mechanisms and implications for training and rehabilitation. Exp. Gerontol. 45, 400–409. 10.1016/j.exger.2010.03.008 20303404 PMC3326066

[B22] Roys M. (2001). Serious stair injuries can be prevented by improved stair design. Appl. Ergon. 32, 135–139. 10.1016/S0003-6870(00)00049-1 11277505

[B23] Startzell J. K. Owens D. A. Mulfinger L. M. Cavanagh P. R. (2000). Stair negotiation in older people: a review. J. Am. Geriatr. Soc. 48, 567–580. 10.1111/j.1532-5415.2000.tb05006.x 10811553

[B24] Svanstrom L. (1974). Falls on stairs: and epidemiological accident study. Scand. J. Soc. Med. 2, 113–120. 10.1177/140349487400200302 4432054

[B25] Tinetti M. E. Speechley M. Ginter S. F. (1988). Risk factors for falls among elderly persons living in the community. N. Engl. J. Med. 319, 1701–1707. 10.1056/NEJM198812293192604 3205267

[B26] Wollacott M. H. Tang P.-F. (1977). Balance control during walking in the older adult: research and its implications. Physi Ther. 77, 646–660. 10.1093/ptj/77.6.646 9184689

[B27] World Health Organisation. (2007). WHO Global Report on Falls Prevention in Older Age. Geneva: World Health Organisation Press. Available online at: https://www.who.int/ageing/publications/Falls_prevention7March.pdf?ua=1

